# Supplementary material for: OpenMM 7: Rapid development of high performance algorithms for molecular dynamics
Source: PLoS Comput Biol. 2017 Jul 26;13(7):e1005659. doi: 10.1371/journal.pcbi.1005659 (PMC5549999; doi:10.1371/journal.pcbi.1005659)
Supplement: S1 Source Code — (ZIP) [file pcbi.1005659.s002.zip › OpenMM-7.0.1-Source/docs/api-python/generated/simtk.openmm.openmm.SerializationNode.html]

SerializationNode — OpenMM 7.0.1.dev-5e86c4f documentation


# SerializationNode¶

*class* `simtk.openmm.openmm.``SerializationNode`(*\*args*, *\*\*kwargs*)¶
:   A SerializationNode stores information about an object during serialization or deserialization.

    When an object is serialized, its SerializationProxy is first called to copy information about the object into a SerializationNode. That information can then be written to the output stream in the desired format.

    When an object is deserialized, the input stream is read and the information is stored into a SerializationNode. The appropriate SerializationProxy is then called to reconstruct the object.

    SerializationNodes are arranged in a tree. There will often be a one-to-one correspondence between objects and SerializationNodes, but that need not always be true. A proxy is free to create whatever child nodes it wants and store information in them using whatever organization is most convenient.

    Each SerializationNode can store an arbitrary set of “properties”, represented as key-value pairs. The key is always a string, while the value may be a string, an int, or a double. If a value is specified using one data type and then accessed as a different data type, the node will attempt to convert the value in an appropriate way. For example, it is always reasonable to call getStringProperty() to access a property as a string. Similarly, you can use setStringProperty() to specify a property and then access it using getIntProperty(). This will produce the expected result if the original value was, in fact, the string representation of an int, but if the original string was non-numeric, the result is undefined.

    `__init__`(*\*args*, *\*\*kwargs*)¶

    Methods

    |  |  |
    | --- | --- |
    | `__init__`(\*args, \*\*kwargs) |  |
    | `createChildNode`((self, ...) | Create a new child node by serializing an object. |
    | `getBoolProperty`((self, name) -> bool) | getBoolProperty(self, name, defaultValue) -> bool |
    | `getChildNode`((self, name) -> SerializationNode) | getChildNode(self, name) -> SerializationNode |
    | `getChildren`(...) | getChildren(self) -> std::vector< OpenMM::SerializationNode,std::allocator< OpenMM::SerializationNode > > & |
    | `getDoubleProperty`((self, name) -> double) | getDoubleProperty(self, name, defaultValue) -> double |
    | `getIntProperty`((self, name) -> int) | getIntProperty(self, name, defaultValue) -> int |
    | `getName`((self) -> std::string const &) | Get the name of this SerializationNode. |
    | `getProperties`((self) -> mapstringstring) | Get a map containing all of this node’s properties. |
    | `getStringProperty`((self, ...) | getStringProperty(self, name, defaultValue) -> std::string const & |
    | `hasProperty`((self, name) -> bool) | Determine whether this node has a property with a particular node. |
    | `setBoolProperty`((self, name, ...) | Set the value of a property, specified as a bool. |
    | `setDoubleProperty`((self, name, ...) | Set the value of a property, specified as a double. |
    | `setIntProperty`((self, name, ...) | Set the value of a property, specified as an int. |
    | `setName`(self, name) | Set the name of this SerializationNode. |
    | `setStringProperty`((self, name, ...) | Set the value of a property, specified as a string. |

    `getName`(*self*) → std::string const &¶
    :   Get the name of this SerializationNode.

    `setName`(*self*, *name*)¶
    :   Set the name of this SerializationNode.

        |  |  |
        | --- | --- |
        | Parameters: | **name** (*string*) – the new name of the SerializationNode |

    `getChildren`(*self*) → std::vector< OpenMM::SerializationNode,std::allocator< OpenMM::SerializationNode > > const¶
    :   getChildren(self) -> std::vector< OpenMM::SerializationNode,std::allocator< OpenMM::SerializationNode > > &

        Get a reference to this node’s child nodes.

    `getChildNode`(*self*, *name*) → SerializationNode¶
    :   getChildNode(self, name) -> SerializationNode

        Get a reference to the child node with a particular name. If there is no child with the specified name, this throws an exception.

        |  |  |
        | --- | --- |
        | Parameters: | **the** (*string*) – name of the child node to get |

    `getProperties`(*self*) → mapstringstring¶
    :   Get a map containing all of this node’s properties.

    `hasProperty`(*self*, *name*) → bool¶
    :   Determine whether this node has a property with a particular node.

        |  |  |
        | --- | --- |
        | Parameters: | **name** (*string*) – the name of the property to check for |

    `getStringProperty`(*self*, *name*) → std::string const¶
    :   getStringProperty(self, name, defaultValue) -> std::string const &

        Get the property with a particular name, specified as a string. If there is no property with the specified name, a default value is returned instead.

        |  |  |
        | --- | --- |
        | Parameters: | - **name** (*string*) – the name of the property to get - **defaultValue** (*string*) – the value to return if the specified property does not exist |

    `setStringProperty`(*self*, *name*, *value*) → SerializationNode¶
    :   Set the value of a property, specified as a string.

        |  |  |
        | --- | --- |
        | Parameters: | - **name** (*string*) – the name of the property to set - **value** (*string*) – the value to set for the property |

    `getIntProperty`(*self*, *name*) → int¶
    :   getIntProperty(self, name, defaultValue) -> int

        Get the property with a particular name, specified as an int. If there is no property with the specified name, a default value is returned instead.

        |  |  |
        | --- | --- |
        | Parameters: | - **name** (*string*) – the name of the property to get - **defaultValue** (*int*) – the value to return if the specified property does not exist |

    `setIntProperty`(*self*, *name*, *value*) → SerializationNode¶
    :   Set the value of a property, specified as an int.

        |  |  |
        | --- | --- |
        | Parameters: | - **name** (*string*) – the name of the property to set - **value** (*int*) – the value to set for the property |

    `getBoolProperty`(*self*, *name*) → bool¶
    :   getBoolProperty(self, name, defaultValue) -> bool

        Get the property with a particular name, specified as a bool. If there is no property with the specified name, a default value is returned instead.

        |  |  |
        | --- | --- |
        | Parameters: | - **name** (*string*) – the name of the property to get - **defaultValue** (*bool*) – the value to return if the specified property does not exist |

    `setBoolProperty`(*self*, *name*, *value*) → SerializationNode¶
    :   Set the value of a property, specified as a bool.

        |  |  |
        | --- | --- |
        | Parameters: | - **name** (*string*) – the name of the property to set - **value** (*bool*) – the value to set for the property |

    `getDoubleProperty`(*self*, *name*) → double¶
    :   getDoubleProperty(self, name, defaultValue) -> double

        Get the property with a particular name, specified as a double. If there is no property with the specified name, a default value is returned instead.

        |  |  |
        | --- | --- |
        | Parameters: | - **name** (*string*) – the name of the property to get - **defaultValue** (*double*) – the value to return if the specified property does not exist |

    `setDoubleProperty`(*self*, *name*, *value*) → SerializationNode¶
    :   Set the value of a property, specified as a double.

        |  |  |
        | --- | --- |
        | Parameters: | - **name** (*string*) – the name of the property to set - **value** (*double*) – the value to set for the property |

    `createChildNode`(*self*, *name*) → SerializationNode¶
    :   Create a new child node by serializing an object. A SerializationProxy is automatically selected based on the object’s type, then invoked to populate the newly created node.

        Note that, while this method is templatized based on the type of object being serialized, the typeid() operator is used to select the proxy. This means the template argument may be a base class, and the correct proxies will still be selected for objects of different subclasses.

        |  |  |
        | --- | --- |
        | Parameters: | - **name** (*string*) – the name of the new node to create - **object** (*T \**) – a pointer to the object to serialize |
        | Returns: | a reference to the newly created node |
        | Return type: | SerializationNode |

    `__delattr__`¶
    :   x.\_\_delattr\_\_(‘name’) <==> del x.name

    `__format__`()¶
    :   default object formatter

    `__getattribute__`¶
    :   x.\_\_getattribute\_\_(‘name’) <==> x.name

    `__hash__`¶

    `__reduce__`()¶
    :   helper for pickle

    `__reduce_ex__`()¶
    :   helper for pickle

    `__sizeof__`() → int¶
    :   size of object in memory, in bytes

    `__str__`¶

High performance molecular simulation on GPUs


### Navigation

- Application Layer
- Library Layer
  - Core Objects
  - Forces
  - Integrators
  - Extras

©2015, Stanford University and the Authors.
|
Powered by Sphinx 1.3.1
& Alabaster 0.7.7
|
Page source
